# Weight change increases the odds of psychological distress in middle age: bidirectional analyses from the Whitehall II Study

**DOI:** 10.1017/S0033291718003379

**Published:** 2018-11-20

**Authors:** Anika Knüppel, Martin J. Shipley, Clare H. Llewellyn, Eric J. Brunner

**Affiliations:** Department of Epidemiology and Public Health, University College London, London WC1E 6BT, UK

**Keywords:** Epidemiology, mood disorder, psychological distress, waist circumference, weight change

## Abstract

**Background:**

Mood disorders and adiposity are major public health challenges. Few studies have investigated the bidirectional association of weight and waist circumference (WC) change with psychological distress in middle age, while taking into account the potential U-shape of the association. The aim of this study was to examine the bidirectional association between psychological distress and categorical change in objectively measured weight and WC.

**Methods:**

We analysed repeated measures (up to 17 522 person-observations in adjusted analyses) of psychological distress, weight and WC from the Whitehall II cohort. Participants were recruited at age 35–55 and 67% male. Psychological distress was assessed using the General Health Questionnaire. We used random-effects regressions to model the association between weight and WC changes and psychological distress, with and without a 5-year lag period.

**Results:**

Psychological distress was associated with weight and WC gain over the subsequent 5 years but not the second 5-year period. Weight gain *and* loss were associated with increased odds for incident psychological distress in models with and without time-lag [odds ratio (OR) for incident psychological distress after 5-year time-lag: loss 1.20, 95% confidence interval (CI) 1.00–1.43; gain>5% 1.20, 95% CI 1.02–1.40]. WC changes were only associated with psychological distress in models without time-lag (OR for incident psychological distress: loss 1.29, 95% CI 1.02–1.64; gain>5% 1.33, 95% CI 1.11–1.58).

**Conclusions:**

Weight gain *and* loss increase the odds for psychological distress compared with stable weight over subsequent 10 years. In contrast, the association between psychological distress and subsequent weight and WC changes was limited to the first 5 years of follow-up.

## Introduction

Mental health and obesity are two major public health challenges in the European region and Western world (WHO, 2015, 2017). An estimated one in three Europeans suffer from symptoms of depression and anxiety at least once in their lifetime, and in the UK, prevalence peaks at middle age (Spiers *et al*., 2011; Steel *et al*., 2014). The prevalence of overweight and obesity also peaks in middle age, at which more than half of adults in developed countries are either overweight or obese (Ng *et al*., 2014).

The nature of the association between obesity and psychological distress is unclear; it could be driven by adiposity increasing the risk of psychological distress, or by psychological distress increasing the risk of obesity. Both pathways may operate (Luppino *et al*., 2010). Studies using an instrumental-variable design have not been able to clarify the dominant direction of the association. As there are no known single-nucleotide polymorphisms for psychological distress, a study used maternal mental health as an instrumental variable for offspring adolescent depression. Findings suggest that adolescent depression was a causal predictor of adult obesity, but not the opposite (Hamer *et al*., 2016). In contrast, two Mendelian randomization studies support adiposity as a cause of depression and psychological distress (Kivimaki *et al*., 2011; Jokela *et al*., 2012). Nevertheless, the nature of the obesity–psychological distress association could differ by age, and there could be common underlying factors that predispose individuals to both conditions (Luppino *et al*., 2010; Kivimaki *et al*., 2011; Jokela *et al*., 2012).

While many studies including middle-aged and older adults found that depression prospectively increases weight and waist circumference (WC) (Forman-Hoffman *et al*., 2007; Sutin and Zonderman, 2012; Brumpton *et al*., 2013; Lasserre *et al*., 2014; Singh *et al*., 2014; de Wit *et al*., 2015; Fezeu *et al*., 2015; Gibson-Smith *et al*., 2016), studies looking at categorical change (e.g. a change in weight status) also found associations with weight loss, suggesting a U-shaped association (Forman-Hoffman *et al*., 2007; de Wit *et al*., 2015; Gibson-Smith *et al*., 2016). Similarly, studies that have looked at the association in the direction from weight changes to depressive symptoms have found both positive *and* negative associations such that weight gain *and* weight loss are associated with increased depressive symptoms (Forman-Hoffman *et al*., 2007; Jackson *et al*., 2014; Khalaila and Litwin, 2014; Singh *et al*., 2014); although in some studies the associations depended on covariates or differed by sex (Forman-Hoffman *et al*., 2007; Jackson *et al*., 2014; Singh *et al*., 2014).

Based on the current evidence, we hypothesized that the prospective relationship between adiposity and psychological distress is both bidirectional and U-shaped. From a public health point of view, establishing the temporal sequence and nature of this relationship in middle age could inform policy makers about targets and timing of interventions and monitoring to reduce the risk for either weight change or mental health issues and thereby increase chances for successful ageing (Brunner *et al*., 2014; Singh-Manoux *et al*., 2014).

Time-lagged analyses help to disentangle temporal sequences of events and shed light on the direction of cause and effect. To our knowledge, two studies in middle-aged adults have investigated the association in this way (Forman-Hoffman *et al*., 2007; Singh *et al*., 2014). However, they were both based on self-reported weight, which could be particularly sensitive to misreporting as participants would require to have tracked their weight over time (Forman-Hoffman *et al*., 2007; Gorber *et al*., 2007; Singh *et al*., 2014). In the light of inconclusive evidence from instrumental analyses and the methodological limitations in studies using time-lagged models, we firstly aim to investigate the bidirectional association of objectively measured weight and WC changes with psychological distress in a cohort of middle-aged British men and women. Secondly, we aim to establish the temporal sequence using time-lagged models in both directions.

## Methods

### Study population

We used data from the Whitehall II cohort study that started in 1985–88. With a response rate of 73%, the initial sample included 10 308 individuals between 35 and 55 years old. From 1985 to 2013, participants were followed up via questionnaire and visited a research clinic for screening (Marmot and Brunner, 2005). The present study has been approved by Joint UCL/UCLH Committee on the Ethics of Human Research and participants have been asked to provide informed consent at every follow-up.

### Measures

#### Relative changes in weight and WC

Weight was measured without shoes and clothing to the nearest 0.1 kg on Soehnle electronic scales every 5 years from 1985–88 to 2012–13. WC was measured by trained staff every 5 years from 1991–93 to 2012–13. Relative change per 5 years was calculated in log percentages [*L*% = 100 × log_e_(*x*_t+1_/*x*_t_), where *x*_t_ and *x*_t+1_ are measurements at time *t* and *t* + 1] (Tornqvist *et al*., 1985) and transformed back to actual percentages for analysis and presentation. Weight/WC loss, moderate and heavy gain were defined as relative change of >−3, >3–5 and >5%, respectively (Stevens *et al*., 2006). Due to the small number of participants losing between −3% and −5% of initial weight, this and the more extreme loss group were combined.

Groups of changers were compared with the stable group, defined as those staying within 3% of their baseline weight or WC.

#### Psychological distress

Psychological distress was measured with the 30-item General Health Questionnaire (GHQ) and defined as scoring ⩾5, based on receiver operating characteristic analysis (Stansfeld and Marmot, 1992; Head *et al*., 2013). The GHQ is a screening questionnaire for non-psychotic psychological distress and elicits depressive and anxiety symptoms such as having lost sleep over worry, or feeling unhappy or depressed (Goldberg, 1972; Stansfeld *et al*., 2002). GHQ caseness has in some studies been described as a common mental disorder (Kivimäki *et al*., 2014; Knüppel *et al*., 2017). The cut-off has been found to have good sensitivity (86.4%) and specificity (87.2%) to measure any mental disorder when compared with a clinical interview in a subsample of the study cohort (Head *et al*., 2013). In sensitivity analyses, associations using higher cut-off scores of ⩾6, ⩾8, ⩾10 were also investigated.

#### Other variables of interest

Covariates were chosen based on literature review and restricted to variables available at data collection phases with psychological distress and weight data. The following covariates were assessed: sex, age, ethnicity (White, South Asian, Black), marital status, (married/cohabiting, single/divorced/widowed), last employment grade level within the civil service (high, intermediate, low), smoking (never, former, current), alcohol intake (none: ⩽1 unit/week, moderate, heavy: ⩾14 units/week), self-reported physical activity (vigorous, moderate and non/mild) (Kumari *et al*., 2004), sleep duration (five categories from ⩽5 to ⩾9 h/day), baseline body mass index class (BMI) (normal: <25 kg/m^2^, overweight, obese: ⩾30 kg/m^2^), weight (kg) or WC (cm), when modelling WC change, diabetes and cardiovascular disease (coronary heart disease and stroke, CVD) based on self-report, clinical examination, Hospital Episode Statistics data or general practitioners contact; information on cancer was based on cancer registration data (Marmot and Brunner, 2005); other longstanding illnesses were self-reported and coded as yes, no or missing; menopausal status was assessed by a question on the age when menstrual bleeding stopped.

Intake of antidepressants, anxiety and antipsychotic medication was self-reported at all phases after phase 4 (1995–99), and from phase 1 (1985–88) to 4 (1995–99) medication intake or current treatment was assumed when doctor diagnosis of depression or anxiety was reported.

### Statistical analysis

We used 5-year cycles of weight/WC and GHQ data across the 25 years of observation, reflecting the time interval between research clinic screenings (Knüppel *et al*., 2017). The observed associations were pooled across these four 5-year cycles (Figure S1). We modelled the association of psychological distress and subsequent relative weight/WC change with a mean number of cycles of 2.1 and 1.6 per individual participant, and relative weight/WC change and subsequent incident psychological distress using multinomial and binomial random-effects logistic regression with a mean number of cycles of 2.5 and 2.0, respectively. This allowed us to model simultaneously, for all cycles combined, the association between the exposure at baseline (*t*_0_) with the outcome at end of the cycle (*t*_1_, 5 years later; *t*_2_, 10 years later), while accounting for within- and between-individual differences (Twisk, 2004). Table S1 depicts the included phases for analyses with and without a time-lag period. As WC was not measured at phase 1 (1985–88), only phases 3 (1991–94) to 11 (2012–13) could be included, resulting in three cycles per analysis direction (Figure S1).

In the first set of analyses, we modelled the association of psychological distress with a relative 5-year weight and WC change (Table 1). In a non-lag model, prevalent psychological distress at each baseline (*t*_0_) was modelled to predict a 5-year change in weight/WC (*t*_1_−*t*_0_), and in a lag model, the same psychological distress prevalence (*t*_0_) was modelled to predict a 5-year change after a 5-year time-lag (*t*_2_−*t*_1_).

**Table 1.** Mode of analyses by direction, exposure and outcome

Direction	Exposure	Outcome	
		Non-lagged	5-year time-lagged
Psychological distress to weight change	Prevalent psychological distress year 0 (*t*_0_)	Relative weight change across 0 to 5 years (*t*_1_−*t*_0_)	Relative weight change across 5–10 years (*t*_1_−*t*_2_)
Weight change to psychological distress	Relative weight change across 0–5 years (*t*_1_−*t*_0_)	Psychological distress at 5 years (*t*_1_), excluding those with psychological distress at 0 years (*t*_0_); referred to as *incident*	Psychological distress at 10 years (*t*_2_), excluding those with psychological distress at 0 years (*t*_0_); referred to as *incident*

Secondly, we modelled the association of a 5-year change in weight and WC and incident psychological distress (Table 1). Incident psychological distress was defined as those having psychological distress, among those who did not have psychological distress at baseline (*t*_0_). In a non-lag model, 5-year change in weight/WC (*t*_1_−*t*_0_) was used to predict incident psychological distress at the cycle when the change was recorded (*t*_1_), and in a lag-model, incident psychological distress after 5 years (*t*_2_) (Table 1, Table S1).

Participants with missing information on ethnicity or ethnicity other than White/South Asian/Black were excluded from analyses. To control for selection bias for the four sets of analysis, the analytical sample was restricted to participants who had three consecutive measures of GHQ and two consecutive measures of weight or WC change. For example, participants with data on weight change from phase 1 (1985–88) to 3 (1991–94) also had to have data on weight changes from phase 3 (1991–94) to 5 (1997–99) and information of psychological distress status at phases 1 (1985–88), 3 (1991–94) and 5 (1997–99). This was done to ensure that participants that contributed to a non-lag model also contributed to the lag model and *vice versa*, and to remove any effect of attrition between the two sets of analyses. Figure S2 shows how the included sample was reached; 18 442 person-observations had sufficient data to be included in analyses between psychological distress and weight change and 12 473 for analyses between psychological distress and WC change. Of these, 4162 and 2623 person-observations, respectively, had psychological distress at each baseline, resulting in 14 280 and 9850 person-observations to be included in the analysis of incident psychological distress. We presented all models with the sample size after exclusion of missing values in covariates (920 person-observations for associations between baseline psychological distress and weight change, 551 and WC change, and 701 for the association between weight change and incident psychological distress at follow-up and 416 between WC change and incident psychological distress at follow-up).

All models were conducted with Stata 14 using the command *xtlogit* and tested for interaction by sex, analysis cycle and age using likelihood ratio tests (StataCorp., 2015). The base regression models were adjusted for sex, age and ethnicity; the fully adjusted models additionally for baseline BMI class, weight/WC, socio-demographic factors, health behaviours, sleep duration and baseline health status (CVD, diabetes and cancer). To investigate the role of health status over the course of follow-up, health status at follow-up (*t*_1_) was administered in time-lagged models. In sensitivity analyses, main analyses were repeated (a) excluding participants with unknown or intake of antidepressants, antipsychotic or anxiety medication at each baseline to account for confounding by treatment of mood disorders; and (b) additionally adjusted for longstanding illnesses in sensitivity analyses, to investigate whether other diseases could confound associations. A posteriori several sensitivity analyses were added: (c) using different cut-off points for GHQ caseness; (d) adjusting additionally for menopausal status and change in status; (e) an analysis for the association of 5-year change in weight and incident psychological distress which excluded participants who had lost weight and reported intentional weight loss based on the question ‘*Are you on a slimming diet now?*’ in a Food Frequency Questionnaire administered at phases 5 (1997–99), 7 (2002–04) and 9 (2007–09). Finally, participants who became GHQ cases 5 years later were compared on the basis whether they had lost or gained weight following the approach used by Gibson-Smith *et al*. (Gibson-Smith *et al*., 2016).

## Results

At phases 1 (1985–88), 3 (1991–94), 5 (1997–99), 7 (2002–04), 9 (2007–09) and 11 (2012–13), the prevalence proportion of psychological distress was 26.8, 21.6, 21.5, 19.9, 14.3 and 16.3% of the eligible sample.

Table 2 shows the comparison of psychological distress cases and non-cases at phase 3. Psychological distress prevalence was higher in younger participants, women, unmarried participants, those who smoked, were less physically active, slept for <7 h/day and those with obesity. Associations were similar at the following phases; non-white ethnicity, lower grade level and CVD were significantly associated with higher psychological distress prevalence at phases 5 (1997–99) and 7 (2002–04) (not depicted). From phase 3 (1991–94) to 5 (1997–99), 11.2% lost weight, 14.6% gained a moderate amount (3–5%) of weight and 37.1% gained a large amount (>5%) of weight; 6.1% decreased their WC, 14.2% increased their WC moderately (3–5%) and 53.7% heavily (>5%). At phase 3 (1991–94), psychological distress prevalence was higher in those who increased their weight or WC.

**Table 2.** Cross-sectional associations between psychological distress and covariates at phase 3 (1991–94)

	No psychological distress (*n* = 3542).		Psychological distress (*n* = 984)		*p*
	Mean/n	±s.d./(%)	Mean/n	±s.d./(%)	
Age	50.0	±6.1	48.9	±5.6	<0.001
Sex					
Women	944	(26.7)	350	(35.6)	<0.001
Ethnic group					0.97
White	3303	(93.3)	918	(93.3)	
South Asian	155	(4.4)	44	(4.5)	
Black	84	(2.4)	22	(2.2)	
Marital status					<0.001
Married/cohabiting	2804	(79.3)	719	(73.1)	
Single/divorced/widowed	734	(20.7)	264	(26.9)	
Last grade level in Civil service†					0.37
Highest	1509	(42.6)	398	(40.4)	
Moderate	1607	(45.4)	471	(47.9)	
Lowest	426	(12.0)	115	(11.7)	
Smoking					0.023
Never-smoker	1690	(50.2)	439	(46.8)	
Ex-smoker	1308	(38.8)	368	(39.2)	
Current smoker	371	(11.0)	132	(14.1)	
Physical activity					<0.001
Non/mild	1202	(33.9)	410	(41.7)	
Moderate	1611	(45.5)	423	(43.0)	
Vigorous	729	(20.6)	151	(15.4)	
Alcohol consumption					0.22
None	718	(20.3)	222	(22.6)	
Moderate	1939	(54.8)	512	(52.0)	
Heavy	881	(24.9)	250	(25.4)	
Sleep duration					<0.001
<7 h/day	811	(22.9)	281	(28.6)	
⩾7 h/day	2729	(77.1)	703	(71.4)	
Weight status (derived using body mass index)					0.032
Normal weight <25 kg/m^2^	1856	(53.6)	524	(55.0)	
Overweight 25–30 kg/m^2^	1345	(38.8)	336	(35.3)	
Obese >30 kg/m^2^	262	(7.6)	92	(9.7)	
Diabetes	80	(2.3)	22	(2.2)	0.97
CVD	87	(2.5)	29	(2.9)	0.39
Cancer	35	(1.0)	8	(0.8)	0.62
Weight change					<0.001
Loss (>−3%)	398	(11.5)	98	(10.3)	
Stable (±3%)	1317	(38.0)	322	(33.7)	
Gain (>3% to ⩽5%)	527	(15.2)	119	(12.5)	
High Gain (>5%)	1225	(35.3)	416	(43.6)	
Waist change					0.028
Loss (>−3%)	192	(6.3)	47	(5.5)	
Stable (±3%)	821	(26.8)	196	(23.0)	
Gain (>3% to ⩽5%)	445	(14.5)	113	(13.3)	
High gain (>5%)	1608	(52.4)	496	(58.2)	

Baseline psychological distress was associated with increased odds for high weight gain (>5%) compared with odds of keeping a stable weight (±3%) in minimally adjusted models and when additionally adjusted for baseline socio-demographic factors, health behaviours, baseline weight, BMI class and disease status (Table 3). Excluding participants with baseline intake of antidepressants, antipsychotic or anxiety medication strengthened the association between psychological distress and weight loss [odds ratio (OR) 0.87, 95% confidence interval (CI) 0.77–0.98, *p* = 0.018, fully adjusted models], and there was no difference after additionally adjusted for any longstanding illnesses. When the analysis was based on a 5-year time-lag, baseline psychological distress was not associated with subsequent weight changes (Table 2).

**Table 3.** Short-term (0–5 years) and long-term (5–10 years) effect of prevalent psychological distress on subsequent weight change

	Outcome: weight change			
	Loss (>−3%)	Stable (±3%)	Gain (>3% to ⩽5%)	High gain (>5%)
Non-time-lagged model (0–5 years change)				
Person-obs.	3030	7657	2358	4477
Psychological distress cases	586	1585	534	1238
OR^a^ (95% CI)	0.93 (0.84–1.04)	Ref.	1.07 (0.96–1.20)	**1.30 (1.20–1.44)**
OR^b^ (95% CI)	**0.89 (0.80–1.00)**	Ref.	1.06 (0.95–1.19)	**1.27 (1.16–1.39)**
5-year time-lagged model (5–10 years change)^c^				
Person-obs.	3737	8109	2095	3581
Psychological distress cases	794	1761	472	916
OR^a^ (95% CI)	0.98 (0.89–1.09)	Ref.	0.96 (0.85–1.08)	1.02 (0.92–1.13)
OR^b^ (95% CI)	0.96 (0.87–1.06)	Ref.	0.95 (0.84–1.07)	1.01 (0.90–1.11)
OR^d^ (95% CI)	0.96 (0.87–1.06)	Ref.	0.95 (0.84–1.07)	1.00 (0.90–1.10)

Bold indicates *p* < 0.05.

a Odds ratios from base model adjusted for age, sex and ethnicity.

b Odds ratios from fully adjusted model: additionally adjusted for marital status, last grade level in civil service, smoking, alcohol intake, physical activity, BMI, weight, diabetes, cardiovascular disease, cancer at baseline.

c Weight change is lagged 5 years after psychological distress assessment (at 0 years).

d Odds ratios additionally adjusted for diabetes, cardiovascular disease, cancer at 5 years.

Compared with person-observations in the stable weight group, participants with gains above 5% of their baseline body weight from 0 to 5 years and losses above 3% had increased odds for incident psychological distress at 5 years and at 10 years (Table 4). This association was independent of baseline socio-demographic factors, health behaviours, weight, BMI class and disease. The associations of weight gain and loss with incident psychological distress at 10 years were marginally attenuated when adjusted for disease at 5 years (*p* = 0.054) and stayed statistically significant when additionally adjusted for longstanding illnesses at baseline and 5 years (*p* = 0.041). Excluding participants taking antidepressants, antipsychotic or anxiety medication at baseline marginally attenuated the association of high weight gain and incident psychological distress 5 years later in fully adjusted models (OR 1.15, 95% CI 0.99–1.33, *p* = 0.061) and the association of weight loss with subsequent incident psychological distress at 10 years (OR 1.19, 95% CI 0.99–1.42, *p* = 0.068, in fully adjusted models).

**Table 4.** Short-term (0–5 years) and long-term (5–10 years) effect of weight change on subsequent incident psychological distress

		Outcome: incident psychological distress						
		Non-time-lagged model (at 5 years)				5-year time-lagged model (at 10 years)^a^		
	Person-obs.	Cases	OR^b^ (95% CI)	OR^c^ (95% CI)	Cases	OR^b^ (95% CI)	OR^c^ (95% CI)	OR^d^ (95% CI)
Loss (>−3%)	2444	322	**1.21 (1.03–1.42)**	**1.19 (1.01–1.40)**	322	**1.21 (1.01–1.44)**	**1.20 (1.00–1.43)**	1.19 (1.00–1.42)
Stable (±3%)	6072	715	Ref.	Ref.	713	Ref.	Ref.	Ref.
Gain (>3% to ⩽5%)	1824	230	0.98 (0.82–1.18)	0.98 (0.82–1.27)	230	1.02 (0.84–1.24)	1.02 (0.84–1.24)	1.02 (0.84–1.24)
High gain (>5%)	3239	519	**1.18 (1.02–1.36)**	**1.17 (1.01–1.35)**	500	**1.21 (1.04–1.42)**	**1.20 (1.02–1.40)**	**1.19 (1.02–1.39)**

a The incident psychological distress is lagged 5 years after weight change (from 0 to 5 years).

b Odds ratios from base model adjusted for age, sex and ethnicity.

c Odds ratios from fully adjusted model: additionally adjusted for marital status, last grade level in civil service, smoking, alcohol intake, physical activity, BMI, weight, diabetes, cardiovascular disease, cancer at baseline.

d Odds ratios additionally adjusted for diabetes, cardiovascular disease, cancer at 5 years.

In sensitivity analyses using cut-off points of 6, 8 and 10 for psychological distress results were generally replicated showing similar ORs at different cut-offs. However, with smaller numbers of participants being classified as psychologically distressed, the association between weight change and psychological distress after a 5-year time lag did not reach statistical significance (see Table S4–S9 for associations with weight change).

Additional adjustment for menopausal status and change in status did not change the conclusions but associations between weight loss (OR 1.28, 95% CI 1.04–1.57) and gain (OR 1.29, 95% CI 1.06–1.59) and incident psychological distress after a lag period were slightly stronger.

Sensitivity analysis excluding participants who lost weight and reported to be on a weight loss diet in a subgroup with available data did not change the association with incident psychological distress (OR for intentional and unintentional weight loss compared with stable weight 1.34, 95% CI 1.02–1.76; OR for unintentional weight loss only 1.37, 95% CI 1.03–1.81).

Associations with WC change in non-lag models were similar to associations with weight change (see Tables S2 and S3). Baseline psychological distress was associated with increased odds for high WC gain (OR 1.22, 95% CI 1.09–1.36) but not after a 5-year time-lag (OR 1.04, 95% CI 0.93–1.16) compared with odds for keeping WC stable. WC loss was associated with increased odds for incident psychological distress at 5 years (OR 1.29, 95% CI 1.02–1.64), but no significant increase at 10 years (OR 1.08, 95% CI 0.83–1.39) and similarly WC gain was associated with an increased odds for incident psychological distress at 5 years (OR 1.33, 95% CI 1.11–1.58) but not at 10 years (OR 1.08, 95% CI 0.90–1.31). There was no association of WC loss with incident psychological distress when participants taking antidepressants, antipsychotic or anxiety medication at baseline were excluded (OR 1.26, 95% CI 0.99–1.60, *p* = 0.065, in models adjusted for age, sex and ethnicity). Additionally, adjusting for any longstanding illnesses did not change results.

There was no evidence for interaction by sex or cycle in any of the modes of analysis. There was an interaction with age (*p* = 0.028) for the association between psychological distress and weight loss, suggesting that baseline psychological distress was mainly associated with decreased odds for weight loss in the younger participants

Both weight loss and weight gain were shown to have an adverse effect on the long-term likelihood of new psychological distress. We compared participants who *lost* weight and became psychological distress cases 5 years later with those who *gained* weight and became psychological distress cases 5 years later (Table 5). People with new psychological distress at follow-up who lost weight on average lost 6.1% (s.d. 3.4) of their initial body weight and those who gained >5% weight gained an average of 9.4% (s.d. 5.0). Compared with weight gainers with psychological distress 5 years later, weight losers were older (*p* < 0.001), physically less active (*p* = 0.058), suffered from more diabetes and CVD at baseline and at the end of the weight change. Participants who lost weight were in higher BMI classes at baseline than those who gained weight. There was no difference in antidepressants, antipsychotic or anxiety medication intake or GHQ summary score, but some differences (*p* < 0.1) in answers to questions in the GHQ. Participants who gained weight were more likely to report having *felt constantly under strain*, participants who lost weight reported more often having not *been able to enjoy your normal day-to-day activities*, having *been taking things hard* and having *felt that life is entirely hopeless*.

**Table 5.** Comparison of person-observations of participants who lost >−3% weight and person-observations of participants who gained >5% weight and suffered from psychological distress psychological distress 5 years later (at 10 years)

	Participants with weight loss (<−3%) and psychological distress (*n* = 337)		Participants with high weight gain (>5%) and psychological distress (*n* = 528)		*p*
	Mean/n	±s.d./(%)	Mean/n	±s.d./(%	
Baseline characteristics					
Women	121	(35.9)	189	(35.8)	0.98
Age	55.1	±9.4	47.7	±8.10	<0.001
Smoking					
Never-smoker	173	(52.1)	277	(53.6)	
Ex-smoker	126	(37.5)	170	(32.9)	
Current smoker	35	(10.4)	70	(13.5)	0.23
Physical activity					
Non/mild	133	(40.1)	191	(36.5)	
Moderate	154	(46.4)	228	(43.6)	
Vigorous	45	(13.6)	104	(19.9)	0.058
Alcohol consumption					
None	79	(23.7)	111	(21.3)	
Moderate	155	(46.6)	273	(52.4)	
Heavy	99	(29.7)	137	(26.3)	0.25
Sleep duration					
<7 h/day	111	(33.0)	166	(31.4)	
⩾7 h/day	225	(67.0)	362	(68.6)	0.49
Disease status at baseline:					
CVD	22	(6.5)	24	(4.6)	0.21
Diabetes	26	(7.7)	17	(3.2)	0.003
Diabetes (self-reported)	15	(4.4)	11	(2.1)	0.047
Cancer	13	(3.9)	6	(1.1)	0.008
Disease status at end of weight change					
CVD	39	(11.6)	39	(7.4)	0.035
Diabetes	40	(11.9)	24	(4.6)	<0.001
Cancer	20	(5.9)	19	(3.6)	0.11
Measures of adiposity					
Baseline BMI class					
Normal	148	(44.2)	291	(55.4)	
Overweight	132	(39.4)	185	(35.2)	
Obese	55	(16.4)	49	(9.3)	<0.001
Weight loss in next 5 years	78	(23.1)	112	(21.2)	0.16
Weight gain in next 5 years	146	(43.3)	205	(38.8)	0.073
Psychological distress details					
Psychological distress at end of weight gain	123	(36.5)	181	(34.3)	0.51
Intake of antidepressants, antipsychotic or anxiety medication					
At baseline	15	(4.5)	19	(3.6)	0.53
At end of weight change	16	(4.8)	32	(4.8)	0.41
At outcome	35	(10.4)	44	(8.4)	0.31
Outcome GHQ					
GHQ score at outcome	10.9	± 5.9	10.7	± 5.7	0.72
Have you recently:					
Question 14 – *felt constantly under strain?*	207	(61.6)	358	(67.8)	0.062
Question 17 – *been able to enjoy your normal day-to-day activities?*^a^	189	(56.1)	259	(49.2)	0.047
Question 18 – *been taking things hard?*	153	(45.8)	208	(39.4)	0.063
Question 25 – *felt that life is entirely hopeless?*	59	(17.5)	66	(12.5)	0.041

a Reverse scored.

## Discussion

This was the first study to investigate the bidirectional association of psychological distress and *objectively measured* weight and WC changes in middle-aged adults using time-lagged analyses. Our findings confirmed the hypothesis of bidirectional associations between psychological distress and relative change in weight and WC short term. The association between prevalent psychological distress and relative change in weight and WC showed a linear trend, while both loss and gain >5% of weight and WC increased the odds of incident psychological distress. When the first 5 years of follow-up were excluded from analysis, weight changes continued to predict incident psychological distress, while the effect of psychological distress on weight change was restricted to the first 5 years. The findings suggest an association in the direction of weight changes to psychological distress.

### Effect of psychological distress on subsequent weight and WC changes

The association between psychological distress and increases in weight and WC in non-lagged models was consistent with previous literature (Forman-Hoffman *et al*., 2007; Lasserre *et al*., 2014; Singh *et al*., 2014; Gibson-Smith *et al*., 2016). We did not find a positive association between psychological distress and weight loss in the main analysis as observed by others (de Wit *et al*., 2015; Gibson-Smith *et al*., 2016). In fact, when including an age interaction and excluding participants with antidepressant, antipsychotic or anxiety medication intake, which might have a pharmacological effect on adiposity, psychological distress was associated with reduced odds of weight loss compared with those not reporting distress, which was stronger at younger ages.

The null association between psychological distress and weight changes in time-lagged models contrasts results of previous studies using this method. Forman-Hoffman *et al*., 2007 and Singh *et al*., 2014 found prevalent depression to be associated with a 2-year weight loss and gain in women after a 2-year time-lag and a 3-year weight gain after a 3-year time-lag, respectively (Forman-Hoffman *et al*., 2007; Singh *et al*., 2014). This discrepancy could be due to the longer period between weight measures and lag length in our study (5 years compared with 2 and 3 years). It is possible that mood disorders only have a short-term effect on weight changes. This association could be explained by dysregulations of the hypothalamic–pituitary–adrenal axis (Peckett *et al*., 2011; Stetler and Miller, 2011) or adverse effects on health behaviours, such as physical activity (Azevedo Da Silva *et al*., 2012).

### Effect of weight and WC changes on incident psychological distress

We found a robust association between weight and WC change (from 0 to 5 years) on odds of an incident psychological distress at 5 years. This finding was in line with previous results from prospective studies (Forman-Hoffman *et al*., 2007; Jackson *et al*., 2014; Khalaila and Litwin, 2014; Singh *et al*., 2014). However, some previous findings depended on adjustment for other factors, and in one study, the association with weight gain was restricted to men (Forman-Hoffman *et al*., 2007; Khalaila and Litwin, 2014; Singh *et al*., 2014).

Weight gain and weight loss (from 0 to 5 years) also increased the chances for mood disorders after a 5-year time-lag (incident psychological distress at 10 years) in models adjusted for covariates at baseline. Although our results differed from the fully adjusted time-lagged models reported by Singh *et al*. (2014) and Forman-Hoffman *et al*. (2007), they are in line with their unadjusted results (Forman-Hoffman *et al*., 2007; Singh *et al*., 2014). In contrast to our analyses, Singh *et al*. (2014) and Forman-Hoffman *et al*. (2007) adjusted for covariates at baseline *and* time-varying factors in fully adjusted models. This could have led to overadjustment as time-varying factors may have collinear associations or act as mediators. In addition, the difference to our findings might be due to the use of self-reported weight. Under-reporting of weight could have resulted in misclassification of participants to the stable weight group, thereby underestimating the true association (Gorber *et al*., 2007).

The association between weight gain and increased chance of incident mood disorder could be explained by biological and psychological mechanisms. Inflammatory markers have been shown to increase with weight gain and elevated marker levels have been found to be associated with increased risk for psychological distress (Fransson *et al*., 2010; Kivimäki *et al*., 2014). Furthermore, weight gain may prompt experiences and perception of weight discrimination, which have been found to explain about 40% of the association of obesity and psychological well-being (Jackson *et al*., 2015).

We tested several potential alternative explanations for the association of weight loss and subsequent psychological distress. We additionally adjusted for health status at 5 years to investigate whether the association was driven by a change in health status over follow-up; while results lost statistical significance the estimates were only marginally attenuated. Our finding was in line with the findings by Jackson *et al*. (2014) who found that the association between weight loss and depressed mood could not be explained by changes in health and major life events (Jackson *et al*., 2014). We excluded participants with intake of antidepressants, antipsychotic or anxiety medication and found small differences in associations. In sensitivity analyses, we tested whether the association was driven by unintentional weight loss. We could not show a difference in the association, suggesting that the association might be independent of weight loss intention. Additional adjustments for longstanding illnesses, menopausal status and status change also did not change the conclusions.

Results from weight loss trials show that lifestyle interventions can improve mood. But the null association between weight loss and depression symptoms within groups suggests that the effect is not fully due to weight loss itself (Fabricatore *et al*., 2011). Outside of a controlled environment, the difficulties surrounding the attainment of weight loss could motivate unhealthy behaviours and negative thoughts (Jackson *et al*., 2014). Compared with a general population, those who are successfully losing weight have been shown to have more depressive symptoms, concerns about health and body shape, engage in binge eating and unhealthy dieting practices (Feller *et al*., 2015). We compared those with incident psychological distress at 10 years that had gained weight to those who had lost weight to elucidate whether they had specific characteristics. Those who lost weight and got depressed were sicker, older and more overweight than those who gained weight. We found little difference in reported symptoms of psychological distress, although a few answers could point to that those who lost weight could have severe symptoms with a severe effect on daily life. Further research is warranted to elucidate the prospective effect of weight loss on mood outside of controlled trial settings.

### Limitations

Limitations need to be considered in the interpretation of our results. Our study was based on a non-representative cohort, which might reduce generalizability of results. For example, the prevalence of obesity in this study (8.0%) was lower than reported in the Health Survey for England in 1993 (13.6%) (Zaninotto *et al*., 2009). Nevertheless, hazard ratios produced in the Whitehall II study have been found to be similar when investigating associations between common risk factors for CVD and CVD risk to those from representative cohorts even though disease incidence and prevalence of exposures differed (Batty *et al*., 2014). Representativeness is further affected by attrition with healthier participants being more likely to remain and be included in this research (Jokela *et al*., 2011).

Mental health was assessed using a population screening tool which may have led to some misclassification of participants. However, the GHQ at a cut-off of 5 was found to be a sensitive and specific measure for any mental disorder in this cohort (Head *et al*., 2013). Further, sensitivity analyses using higher cut points produced similar associations. Psychological distress has been found to be recurrent in 36% of cases in Whitehall II; it cannot be ruled out that associations might differ by number of caseness episodes (Jokela *et al*., 2011). Moreover, the 5-year period between screening phases could not be investigated.

Despite the wide range of covariates included in the models, residual confounding could still be present. Hypothetically, there may be pleiotropic genetic predisposition for both psychological distress and higher or lower adiposity. Furthermore, use of antidepressants, antipsychotic or anxiety medication could not be directly identified in data collection up to phase 4 and was inferred from self-reported doctor diagnoses of depression and anxiety.

Finally, the relatively long period between screening phases did not fully allow for the role of *short-term* changes in weight, WC and psychological distress to be investigated.

The present study examined bidirectional associations between psychological distress and adiposity across 10 years. In mid-life, it appears there are long-term adverse effects of weight loss and substantive weight gain, over 5%, on psychological distress. Conversely, low mood was associated with weight and WC gain in the short term (0–5 years), but this effect was not evident in the longer term. Our findings suggest that monitoring of weight changes in both directions could help identify persons at risk of mood disturbances in middle age. Interventions encouraging the maintenance of weight could have a protective effect on mental health.

## Supplementary material

For supplementary material accompanying this paper visit https://doi.org/10.1017/S0033291718003379. click here to view supplementary material
