# Supplementary material for: Weight change increases the odds of psychological distress in middle age: bidirectional analyses from the Whitehall II Study
Source: Psychol Med. 2018 Nov 20;49(15):2505–14. doi: 10.1017/S0033291718003379 (PMC6805986; doi:10.1017/S0033291718003379)
Supplement: Supplementary file 1 [file S0033291718003379sup001.docx]

**Weight change increases the odds of psychological distress in middle age: bidirectional analyses from the Whitehall II Study**

Anika Knüppel^1^*, Martin J. Shipley^1^, Clare H. Llewellyn^1^, Eric J. Brunner^1^

^1^Department of Epidemiology and Public Health, University College London, London WC1E 6BT, UK

*Corresponding author: anika.knuppel@ndph.ox.ac.uk


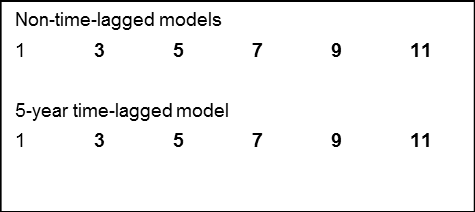


Figure S1 Modes of analyses using a cycle approach for non-lagged and 5-year time-lagged models

Numbers indicate study collection phases. Arrows present analysis cycles from exposure to outcome phase with the number of arrows representing the number of cycles in the analysis. Bold numbers represent those with data collection of waist circumference.

Participants at Phases 1 / 3 / 5 / 7 / 9 / 11, n=10308 / 8815 / 7870 / 6967 / 6761 / 6308

Participants with sufficient data on psychological distress:

Phases 1 / 3 / 5 / 7 / 9 /11, n= 10023 / 8215 / 6983 / 6667 / 6516 / 6102

Excluded: ethnicity unknown or not White / South Asian / Black,
n= 171/104 / 66 / 65 / 63 / 52

Participants at Phases 1 / 3 / 5 / 7 / 9 / 11, n= 10137 / 8711 / 7804 / 6902 / 6698 / 6256

Excluded: Psychological distress missing, n= 114 / 496 / 821 / 235 / 182 / 154

Excluded: waist circumference not measured,
n= n.a. / 484 / 2083 / 408 / 498 / 631

Excluded: weight not measured,
n= 10 / 394 / 1516 / 411 / 496 / 634

Participants with sufficient weight change data:

Phases 1 / 3 / 5 / 7 / 9 /11,
n= 7911 / 5336 / 4748 / 5546 / 5257 / n.a.

Participants with sufficient waist change data:

Phases 1 / 3 / 5 / 7 / 9 /11,
n= n.a. / 4748 / 4280 / 5548 / 5259 / n.a.

Participants with sufficient data to be included in all analyses modes using weight:

Phases 1 / 3 / 5 / 7 / 9 /11,
n= 5091 / 4422 / 4170 / 4759 / n.a. / n.a.
Incidence models:

n= 3727 / 3467 / 3274 / 3812 / n.a. / n.a.

Participants with sufficient data to be included in all analyses modes using waist circumference: Phases 1 / 3 / 5 / 7 / 9 /11,
n= n.a. / 3955 / 3756 / 4762 / n.a. / n.a.

Incidence models:

n= n.a. / 3095 / 2941 / 3814 / n.a. / n.a

**Figure S2 Sample Inclusion of person observations by phase**Abbreviations: n.a. not available

**Table S 1 Data collection phases included in non-lagged and 5-year time-lagged analysis cycles**

| **Direction** | **Exposure** | **Outcome non-lagged** | **5-year time-lagged** |
| --- | --- | --- | --- |
| **Psychological distress 🡪 Weight change** | | | |
|  | Phase 1: Psychological distress | Phase 1 to 3: weight change | Phase 3 to 5: weight change |
|  | Phase 3: Psychological distress | Phase 3 to 5: weight change | Phase 5 to 7: weight change |
|  | Phase 5: Psychological distress | Phase 5 to 7: weight change | Phase 7 to 9: weight change |
|  | Phase 7: Psychological distress | Phase 7 to 9: weight change | Phase 9 to 11: weight change |
|  | Prevalent psychological distress year 0 (t_0_) | Change across 0 to 5 years (t_1_-t_0_) | Change across 5 to 10 years (t_1_-t_2_) |
| **Weight change 🡪 incident psychological distress** | | | |
|  | Phase 1 to 3: weight change | Phase 3: incident psychological distress | Phase 5: incident psychological distress |
|  | Phase 3 to 5: weight change | Phase 5: incident psychological distress | Phase 7: incident psychological distress |
|  | Phase 5 to 7: weight change | Phase 7: incident psychological distress | Phase 9: incident psychological distress |
|  | Phase 7 to 9: weight change | Phase 9: incident psychological distress | Phase 11: incident psychological distress |
|  | Change across 0 to 5 years (t_1_-t_0_) | Incident psychological distress at 5 years (t_1_) | Incident psychological distress at 10 years (t_2_) |

**Table S2 Short-term (0 to 5 year) and long-term (5 to 10 year) effect of prevalent psychological distress on subsequent WC change**

|  | **Outcome: WC change** | | | |
| --- | --- | --- | --- | --- |
|  | Loss (> -3%) | Stable (± 3%) | Gain (> 3% to ≤ 5%) | High Gain (> 5%) |
| Non-time-lagged model (0 to 5 year change) | | | | |
| Person-obs. | 1526 | 4367 | 1773 | 4256 |
| psychological distress cases | 291 | 822 | 357 | 1018 |
| OR^a^ (95%-CI) | 1.00 (0.86, 1.17) | Ref. | 1.07 (0.93, 1.23) | **1.25 (1.12, 1.40)** |
| OR^b^ (95%-CI) | 0.97 (0.82, 1.14) | Ref. | 1.07 (0.92, 1.23) | **1.22 (1.09, 1.36)** |
| 5-year time-lagged model (5 to 10 year change)^c^ | | | | |
| Person-obs. | 1941 | 4812 | 1732 | 3437 |
| psychological distress cases | 407 | 976 | 348 | 757 |
| OR^a^ (95%-CI) | 1.02 (0.89, 1.16) | Ref. | 0.97 (0.84, 1.11) | 1.04 (0.93, 1.16) |
| OR^b^ (95%-CI) | 1.00 (0.87, 1.14) | Ref. | 0.96 (0.84, 1.11) | 1.04 (0.93, 1.16) |
| OR^d^ (95%-CI) | 1.00 (0.87, 1.15) | Ref. | 0.96 (0.84, 1.11) | 1.04 (0.93, 1.16) |
|  |  |  |  |  |

^a^ Odds ratios from base model adjusted for age, sex and ethnicity.

^b^ Odds ratios from fully adjusted model: additionally adjusted for marital status, last grade level in civil service, smoking, alcohol intake, physical activity, BMI, WC, diabetes, cardiovascular disease, cancer at baseline

^c^ WC change is lagged 5 years after psychological distress assessment ( at 0 years).

^d^ Odds ratios additionally adjusted for diabetes, cardiovascular disease, cancer at 5 years.

**Table S3 Short-term (0 to 5 year) and long-term (5 to 10 year) effect of WC change on subsequent incident psychological distress**

|  |  | **Outcome: Incident psychological distress** | | | | | | |  |
| --- | --- | --- | --- | --- | --- | --- | --- | --- | --- |
|  |  | Non-time-lagged model (at 5 years) | | |  |  | 5-year time-lagged model (at 10 years)^a^ | | |
|  | Person-Obs. | cases | OR^b^ (95%-CI) | OR^c^ (95%-CI) |  | cases | OR^b^ (95%-CI) | OR^c^ (95%-CI) | OR^d^ (95%-CI) |
|  |  |  |  |  |  |  |  |  |  |
| Loss (> -3%) | 1,235 | 152 | **1.31 (1.03, 1.66)** | **1.29 (1.02, 1.64)** |  | 145 | 1.08 (0.84, 1.38) | 1.08 (0.83, 1.39) | 1.07 (0.83, 1.38) |
| Stable (± 3%) | 3,545 | 370 | Ref. | Ref. |  | 402 | Ref. | Ref. | Ref. |
| Gain (> 3% to ≤ 5%) | 1,416 | 166 | 1.12 (0.89, 1.41) | 1.12 (0.89, 1.40) |  | 145 | 0.82 (0.64, 1.06) | 0.82 (0.64, 1.05) | 0.82 (0.64, 1.05) |
| High Gain (> 5%) | 3,238 | 493 | **1.33 (1.12, 1.59)** | **1.33 (1.11, 1.58)** |  | 440 | 1.10 (0.92, 1.33) | 1.08 (0.90, 1.31) | 1.08 (0.89, 1.30) |
|  |  |  |  |  |  |  |  |  |  |

^a^ The incident psychological distress is lagged 5 years after WC change (from 0 to 5 years).

^b^ Odds ratios from base model adjusted for age, sex and ethnicity.

^c^ Odds ratios from fully adjusted model: additionally adjusted for marital status, last grade level in civil service, smoking, alcohol intake, physical activity, BMI, WC, diabetes, cardiovascular disease, cancer at baseline

^d^ Odds ratios additionally adjusted for diabetes, cardiovascular disease, cancer at 5 years.

Table S 4 Short-term (0 to 5 year) and long-term (5 to 10 year) effect of prevalent psychological distress (GHQ ≥ 6) on subsequent weight change

|  | **Outcome: Weight change** | | | |
| --- | --- | --- | --- | --- |
|  | Loss (> -3%) | Stable (± 3%) | Gain (> 3% to ≤ 5%) | High Gain (> 5%) |
| Non-time-lagged model (0 to 5 year change) | | | | |
| Person-obs. | 3030 | 7657 | 2358 | 4477 |
| Psychological distress (GHQ ≥ 6) | 503 | 1,375 | 471 | 1,077 |
| OR^a^ (95%-CI) | 0.93 (0.82, 1.04) | Ref. | 1.09 (0.97, 1.23) | **1.30 (1.18, 1.43)** |
| OR^b^ (95%-CI) | 0.89 (0.79, 1.00) | Ref. | 1.08 (0.96, 1.22) | **1.26 (1.14, 1.39)** |
| 5-year time-lagged model (5 to 10 year change)^c^ | | | | |
| Person-obs. | 3737 | 8109 | 2095 | 3581 |
| Psychological distress (GHQ ≥ 6) | 683 | 1,523 | 419 | 801 |
| OR^a^ (95%-CI) | 0.98 (0.88, 1.09) | Ref. | 0.99 (0.87, 1.12) | 1.02 (0.91, 1.13) |
| OR^b^ (95%-CI) | 0.95 (0.86, 1.06) | Ref. | 0.98 (0.86, 1.11) | 1.00 (0.90, 1.11) |
| OR^d^ (95%-CI) | 0.95 (0.86, 1.06) | Ref. | 0.97 (0.86, 1.10) | 1.00 (0.90, 1.11) |
|  |  |  |  |  |

^a^ Odds ratios from base model adjusted for age, sex and ethnicity**.**

^b^ Odds ratios from fully adjusted model: additionally adjusted for marital status, last grade level in civil service, smoking, alcohol intake, physical activity, BMI, weight, diabetes, cardiovascular disease, cancer at baseline

^c^ Weight change is lagged 5 years after psychological distress assessment (at 0 years).
^d^ Odds ratios additionally adjusted for diabetes, cardiovascular disease, cancer at 5 years

Table S 5 Short-term (0 to 5 year) and long-term (5 to 10 year) effect of weight change on subsequent incident psychological distress (GHQ ≥ 6)

|  |  | **Outcome: Incident psychological distress (GHQ ≥ 6)** | | | | | | |  |
| --- | --- | --- | --- | --- | --- | --- | --- | --- | --- |
|  |  | Non-time-lagged model (at 5 years) | | |  |  | 5-year time-lagged model (at 10 years)^a^ | | |
|  | Person-Obs. | cases | OR^b^ (95%-CI) | OR^c^ (95%-CI) |  | cases | OR^b^ (95%-CI) | OR^c^ (95%-CI) | OR^d^ (95%-CI) |
|  |  |  |  |  |  |  |  |  |  |
| Loss (> -3%) | 2,444 | 273 | **1.25 (1.05, 1.48)** | **1.24 (1.04, 1.47)** |  | 269 | **1.20 (0.99, 1.45)** | 1.19 (0.98, 1.44) | 1.18 (0.98, 1.43) |
| Stable (± 3%) | 6,072 | 592 | Ref. | Ref. |  | 605 | Ref. | Ref. | Ref. |
| Gain (> 3% to ≤ 5%) | 1,824 | 187 | 0.95 (0.78, 1.15) | 0.95 (0.78, 1.15) |  | 195 | 1.01 (0.82, 1.25) | 1.02 (0.82, 1.26) | 1.02 (0.82, 1.26) |
| High Gain (> 5%) | 3,239 | 439 | **1.18 (1.01, 1.38)** | **1.17 (1.01, 1.37)** |  | 407 | 1.12 (0.94, 1.33) | 1.10 (0.93, 1.31) | 1.10 (0.92, 1.30) |
|  |  |  |  |  |  |  |  |  |  |

^a^ The incident psychological distress is lagged 5 years after weight change (from 0 to 5 years).

^b^ Odds ratios from base model adjusted for age, sex and ethnicity.

^c^ Odds ratios from fully adjusted model: additionally adjusted for marital status, last grade level in civil service, smoking, alcohol intake, physical activity, BMI, weight, diabetes, cardiovascular disease, cancer at baseline

^d^ Odds ratios additionally adjusted for diabetes, cardiovascular disease, cancer at 5 years.

Table S 6 Short-term (0 to 5 year) and long-term (5 to 10 year) effect of prevalent psychological distress (GHQ ≥ 8) on subsequent weight change

|  | **Outcome: Weight change** | | | |
| --- | --- | --- | --- | --- |
|  | Loss (> -3%) | Stable (± 3%) | Gain (> 3% to ≤ 5%) | High Gain (> 5%) |
| Non-time-lagged model (0 to 5 year change) | | | | |
| Person-obs. | 3030 | 7657 | 2358 | 4477 |
| Psychological distress (GHQ ≥ 8) | 376 | 1,048 | 359 | 836 |
| OR^a^ (95%-CI) | 0.90 (0.79, 1.02) | Ref. | 1.08 (0.95, 1.24) | **1.29 (1.16, 1.44)** |
| OR^b^ (95%-CI) | **0.85 (0.75, 0.98)** | Ref. | 1.07 (0.94, 1.22) | **1.24 (1.12, 1.38)** |
| 5-year time-lagged model (5 to 10 year change)^c^ | | | | |
| Person-obs. | 3737 | 8109 | 2095 | 3581 |
| Psychological distress (GHQ ≥ 8) | 527 | 1,169 | 321 | 602 |
| OR^a^ (95%-CI) | 0.98 (0.87, 1.10) | Ref. | 0.98 (0.86, 1.13) | 0.98 (0.87, 1.10) |
| OR^b^ (95%-CI) | 0.95 (0.84, 1.07) | Ref. | 0.97 (0.85, 1.12) | 0.96 (0.85, 1.08) |
| OR^d^ (95%-CI) | 0.95 (0.85, 1.07) | Ref. | 0.97 (0.84, 1.11) | 0.96 (0.85, 1.08) |
|  |  |  |  |  |

^a^ Odds ratios from base model adjusted for age, sex and ethnicity**.**

^b^ Odds ratios from fully adjusted model: additionally adjusted for marital status, last grade level in civil service, smoking, alcohol intake, physical activity, BMI, weight, diabetes, cardiovascular disease, cancer at baseline

^c^ Weight change is lagged 5 years after psychological distress assessment (at 0 years).
^d^ Odds ratios additionally adjusted for diabetes, cardiovascular disease, cancer at 5 years

Table S 7 Short-term (0 to 5 year) and long-term (5 to 10 year) effect of weight change on subsequent incident psychological distress (GHQ ≥ 8)

|  |  | **Outcome: Incident psychological distress (GHQ ≥ 8)** | | | | | | |  |
| --- | --- | --- | --- | --- | --- | --- | --- | --- | --- |
|  |  | Non-time-lagged model (at 5 years) | | |  |  | 5-year time-lagged model (at 10 years)^a^ | | |
|  | Person-Obs. | cases | OR^b^ (95%-CI) | OR^c^ (95%-CI) |  | cases | OR^b^ (95%-CI) | OR^c^ (95%-CI) | OR^d^ (95%-CI) |
|  |  |  |  |  |  |  |  |  |  |
| Loss (> -3%) | 2,444 | 186 | 1.12 (0.91, 1.37) | 1.11 (0.91, 1.36) |  | 197 | 1.24 (1.00, 1.55) | 1.24 (0.99, 1.55) | 1.23 (0.99, 1.54) |
| Stable (± 3%) | 6,072 | 438 | Ref. | Ref. |  | 431 | Ref. | Ref. | Ref. |
| Gain (> 3% to ≤ 5%) | 1,824 | 141 | 0.96 (0.77, 1.20) | 0.96 (0.77, 1.20) |  | 140 | 1.01 (0.79, 1.29) | 1.01 (0.79, 1.30) | 1.01 (0.79, 1.30) |
| High Gain (> 5%) | 3,239 | 328 | 1.16 (0.98, 1.38) | 1.16 (0.97, 1.37) |  | 313 | 1.20 (0.99, 1.46) | 1.18 (0.97, 1.44) | 1.18 (0.97, 1.43) |
|  |  |  |  |  |  |  |  |  |  |

^a^ The incident psychological distress is lagged 5 years after weight change (from 0 to 5 years).

^b^ Odds ratios from base model adjusted for age, sex and ethnicity.

^c^ Odds ratios from fully adjusted model: additionally adjusted for marital status, last grade level in civil service, smoking, alcohol intake, physical activity, BMI, weight, diabetes, cardiovascular disease, cancer at baseline

^d^ Odds ratios additionally adjusted for diabetes, cardiovascular disease, cancer at 5 years.

Table S 8 Short-term (0 to 5 year) and long-term (5 to 10 year) effect of prevalent psychological distress (GHQ >10) on subsequent weight change

|  | **Outcome: Weight change** | | | |
| --- | --- | --- | --- | --- |
|  | Loss (> -3%) | Stable (± 3%) | Gain (> 3% to ≤ 5%) | High Gain (> 5%) |
| Non-time-lagged model (0 to 5 year change) | | | | |
| Person-obs. | 3030 | 7657 | 2358 | 4477 |
| Psychological distress (GHQ ≥ 10) | 286 | 801 | 277 | 635 |
| OR^a^ (95%-CI) | 0.89 (0.77, 1.04) | Ref. | 1.09 (0.94, 1.26) | **1.27 (1.13, 1.43)** |
| OR^b^ (95%-CI) | **0.86 (0.74, 1.00)** | Ref. | 1.08 (0.93, 1.25) | **1.21 (1.08, 1.37)** |
| 5-year time-lagged model (5 to 10 year change)^c^ | | | | |
| Person-obs. | 3737 | 8109 | 2095 | 3581 |
| Psychological distress (GHQ ≥ 10) | 394 | 891 | 258 | 456 |
| OR^a^ (95%-CI) | 0.95 (0.84, 1.09) | Ref. | 1.05 (0.90, 1.22) | 0.98 (0.86, 1.12) |
| OR^b^ (95%-CI) | 0.93 (0.81, 1.06) | Ref. | 1.03 (0.88, 1.20) | 0.96 (0.84, 1.09) |
| OR^d^ (95%-CI) | 0.93 (0.81, 1.06) | Ref. | 1.03 (0.88, 1.20) | 0.96 (0.84, 1.09) |
|  |  |  |  |  |

^a^ Odds ratios from base model adjusted for age, sex and ethnicity**.**

^b^ Odds ratios from fully adjusted model: additionally adjusted for marital status, last grade level in civil service, smoking, alcohol intake, physical activity, BMI, weight, diabetes, cardiovascular disease, cancer at baseline

^c^ Weight change is lagged 5 years after psychological distress assessment (at 0 years).
^d^ Odds ratios additionally adjusted for diabetes, cardiovascular disease, cancer at 5 years

Table S 9 Short-term (0 to 5 year) and long-term (5 to 10 year) effect of weight change on subsequent incident psychological distress (GHQ >10)

|  |  | **Outcome: Incident psychological distress (GHQ ≥ 10)** | | | | | | |  |
| --- | --- | --- | --- | --- | --- | --- | --- | --- | --- |
|  |  | Non-time-lagged model (at 5 years) | | |  |  | 5-year time-lagged model (at 10 years)^a^ | | |
|  | Person-Obs. | cases | OR^b^ (95%-CI) | OR^c^ (95%-CI) |  | cases | OR^b^ (95%-CI) | OR^c^ (95%-CI) | OR^d^ (95%-CI) |
|  |  |  |  |  |  |  |  |  |  |
| Loss (> -3%) | 2,444 | 156 | 1.39 (1.11, 1.74) | **1.37 (1.09, 1.72)** |  | 148 | 1.24 (0.97, 1.59) | 1.23 (0.96, 1.58) | 1.23 (0.96, 1.57) |
| Stable (± 3%) | 6,072 | 305 | Ref. | Ref. |  | 321 | Ref. | Ref. | Ref. |
| Gain (> 3% to ≤ 5%) | 1,824 | 97 | 0.94 (0.72, 1.22) | 0.94 (0.72, 1.22) |  | 103 | 1.00 (0.76, 1.32) | 1.01 (0.76, 1.33) | 1.01 (0.76, 1.33) |
| High Gain (> 5%) | 3,239 | 241 | **1.23 (1.00, 1.50)** | 1.21 (0.99, 1.48) |  | 235 | 1.20 (0.97, 1.50) | 1.20 (0.96, 1.49) | 1.19 (0.96, 1.48) |
|  |  |  |  |  |  |  |  |  |  |

^a^ The incident psychological distress is lagged 5 years after weight change (from 0 to 5 years).

^b^ Odds ratios from base model adjusted for age, sex and ethnicity.

^c^ Odds ratios from fully adjusted model: additionally adjusted for marital status, last grade level in civil service, smoking, alcohol intake, physical activity, BMI, weight, diabetes, cardiovascular disease, cancer at baseline

^d^ Odds ratios additionally adjusted for diabetes, cardiovascular disease, cancer at 5 years.
